# Knowledge, Skills, and Experience With Technology in Relation to Nutritional Intake and Physical Activity Among Older Adults at Risk of Falls: Semistructured Interview Study

**DOI:** 10.2196/52575

**Published:** 2024-05-08

**Authors:** Julie Kikkenborg, Emma Magelund, Maria Silke Riise, Lars Kayser, Rikke Terp

**Affiliations:** 1 Department of Clinical Physiology & Nuclear Medicine Rigshospitalet Copenhagen Denmark; 2 Section of Health Service Research Department of Public Health University of Copenhagen Copenhagen Denmark; 3 Department of Internal Medicine Herlev and Gentofte Hospital Copenhagen University Hospital Hellerup Denmark

**Keywords:** eHealth, self-management, fall prevention, older adults, physical activity, nutritional intake, Readiness and Enablement Index for Health Technology, READHY, social support, support, management, fall, nutrition, diet, qualitative study, malnutrition, physical inactivity, injury, injuries, food, food intake, nutritional needs, outpatient clinic, social network, mobile phone

## Abstract

**Background:**

More than one-third of older adults (aged ≥65 y) experience falls every year. The prevalent modifiable risk factors for falling are malnutrition and physical inactivity, among others. The involvement of older adults in the prevention of falls can decrease injuries, hospitalizations, and dependency on health care professionals. In this regard, eHealth can support older adults’ self-management through more physical activity and adequate food intake. eHealth must be tailored to older adults’ needs and preferences so that they can reap its full benefits. Therefore, it is necessary to gain insight into the knowledge, skills, and mindset of older adults living at home who are at risk of falls regarding eHealth.

**Objective:**

This qualitative study aims to explore older adults’ use of everyday digital services and technology and how they acquire knowledge about and manage their nutritional intake and physical activity in relation to their health.

**Methods:**

Semistructured interviews were conducted with 15 older adults (n=9, 60% women; n=6, 40% men; age range 71-87 y) who had all experienced falls or were at risk of falling. These individuals were recruited from a geriatric outpatient clinic. The interviews were analyzed using deductive content analysis based on a modification of the Readiness and Enablement Index for Health Technology framework.

**Results:**

The qualitative data showed that the informants’ social networks had a positive impact on their self-management, use of technology, and mindset toward nutritional intake and physical activity. Although the informants generally lived active lives, they all lacked knowledge about how their food intake influenced their physical health, including their risk of falling. Another finding was the large diversity in the use of technology among the informants, which was related to their mindset toward technology.

**Conclusions:**

Older adults can use technology for everyday purposes, but some need additional introduction and support to be able to use it for managing their health. They also need to learn about the importance of proper nutritional intake and physical activity in preventing falls. Older adults need a more personalized introduction to technology, nutrition, and physical activity in their contact with health professionals.

## Introduction

### Background

Among older adults, falls are common occurrences, with one-third of the population aged ≥65 years experiencing falls every year [1]. Falls are among the major causes of mortality and morbidity in older adults [2,3], and they contribute to social and economic costs as well as create a dependency on health care professionals [4]. A reduction in the incidence of falls would increase older adults’ quality of life [5], relieve the pressure on health care professionals by reducing hospitalizations [6], and reduce health care system costs [4].

Malnutrition and physical inactivity are well-known modifiable behavioral risk factors for falls in older adults [7-9]. Older adults who are malnourished have a 45% higher risk of falling at least once [7]. Increased daily activity and moderate strength and intensity training 3 times a week can reduce the risk of falling by 30% [8]. Therefore, behavior change regarding nutritional intake and physical activity is important to prevent falls, which is also recommended in international clinical guidelines [8,10]. Behavior change in individuals is influenced by their self-management abilities [11,12].

eHealth, defined as “an emerging field at the intersection of medical informatics, public health, and business, referring to health services and information delivered or enhanced through the Internet and related technologies” [13], is often considered an effective tool for supporting self-management [14]. It may serve as an important “copilot” for older adults to increase their awareness of their nutritional needs and motivate them to engage in higher levels of physical activity [15-17]. To ensure that older adults adopt eHealth and find it useful, their level of knowledge, skills, and experience with eHealth must be addressed when they are introduced to it [18,19]. This includes the level of digital health literacy because it is a determinant considered important for technology adoption [20] and self-management [14].

Another important factor is the existence of social support from relatives and friends to successfully find and understand health information [21,22]. Support and encouragement from health care professionals are also essential for the sustainable adoption of health information and eHealth [22,23].

Older adults’ perceptions and mindset may affect their engagement with health initiatives [24,25]. We have previously shown that older adults’ perceptions and mindset affect their use of technology [6] and self-management [26]. Among older hospitalized patients, we found that a lack of awareness regarding meeting their nutritional needs was related to limited knowledge of their nutritional requirements and the impacts of food intake on their physical function [26]. Consequently, many older adults may not eat adequately [26].

The use of eHealth, the existence of social relations, and the capability to manage one’s own condition, as well as perceptions and mindset, are all intermingled factors and important to include to obtain a well-functioning sociotechnical ecosystem that enables healthy behavior. In this study, we explore these aspects in the context of nutritional intake and physical activity in older adults living at home who are at risk of falling.

### Objectives

The aim of this study is to explore older adults’ use of everyday digital services and technology and how they acquire knowledge about, and manage, their nutritional intake and physical activity in relation to their health. These data may help differentiate among users and address specific gaps in relation to knowledge, skills, and mindset. The findings will provide insight into whether eHealth may be a feasible approach to enable and engage older adults living at home who are at risk of falling.

## Methods

### Overview

This is an explorative qualitative study based on semistructured interviews, which were analyzed using content analysis with a deductive approach. The study is part of a larger research program exploring how, through the use of eHealth, we can optimize older adults’ self-management regarding nutritional intake and physical activity to prevent functional decline and reduce the risk of falling. An intervention outlining how health care professionals can assist and support older adults in self-management through the use of eHealth will be designed, developed, and tested. The findings of the study reported in this paper will inform the design of the intervention.

### Participants and Setting

All informants were recruited through convenience sampling from a geriatric outpatient clinic in a university hospital in the capital region of Denmark. An exercise physiologist employed at the clinic obtained informed consent, allowing the first authors (JK, EM, and MSR) to contact and inform the informants before their inclusion in the study. The inclusion criteria were age ≥65 years, good comprehension of the Danish language, and being at risk of falling. The exclusion criterion was the inability to provide informed consent because of cognitive impairment. Before being interviewed, 16 informants were contacted by telephone by one of the first authors to inform them about the study and arrange the interviews. Of these 16 informants, 1 (6%) retracted their earlier decision to participate after the telephone call. Of the 15 informants, 12 (80%) were interviewed in February and March 2022; the remaining 3 (20%; all male informants to ensure information power by gaining varied experiences from both sexes [27]) were interviewed in February and March 2023 [27].

### Theoretical Framework

**Figure 1:**
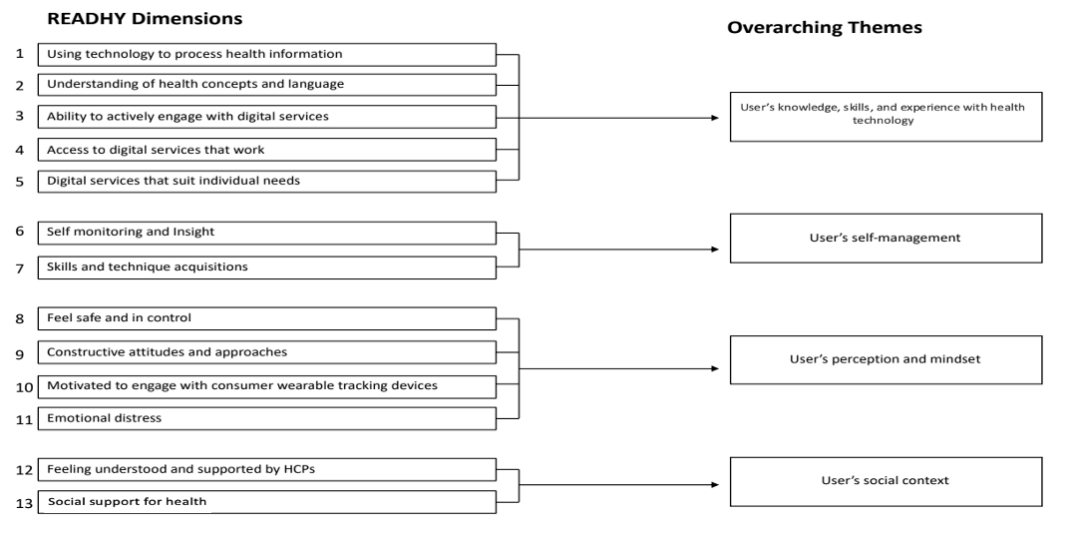
Aggregated themes. HCP: health care professional; READHY: Readiness and Enablement Index for Health Technology.

This study is based on a sociotechnical perspective, meaning that technologies are seen as actors that interact with the users in specific contexts instead of being passive tools [28]. To explore our informants’ ability to engage with eHealth, we used the Readiness and Enablement Index for Health Technology (READHY) as the theoretical framework for the interview guide and analysis [22]. The framework can be used to describe individuals’ readiness for, and enablement by, eHealth. It consists of 13 dimensions within 3 main themes: self-management, social support, and digital health literacy [18]. In this study, we applied the modified READHY framework proposed by M Blaauwhof (personal communication, November 2020). The 13 dimensions were aggregated into the following four main categories: (1) the user’s knowledge, skills, and experience with eHealth; (2) the user’s self-management; (3) the user’s perception and mindset; and (4) the user’s social context (Figure 1; M Blaauwhof, personal communication, November 2020). The main categories were used to understand how eHealth could be applied to support older adults’ health behaviors regarding nutritional intake and physical activity.

### Data Collection

Data were collected from 15 semistructured interviews using an interview guide (Multimedia Appendix 1) with open-ended questions about nutritional intake and physical activity, self-management, social context, and the use of technology. All interviews were conducted by the first authors and took place in the informants’ homes. A minimum of 2 of the 3 first authors were present during the interviews. Of the 15 informants, 4 (27%) had spouses present during the interviews. The interviews lasted from 23 to 60 minutes, with an average of 42 (SD 12) minutes.

### Data Analysis

The interviews were recorded using Microsoft Teams, transcribed by the first authors, and analyzed using deductive content analysis [29] based on the 4 categories from the modified READHY framework. The codes for the deductive content analysis were identified by the first authors by dividing the 4 categories from the modified READHY framework into subcategories and afterward into codes. The first authors coded the interviews using the data management software program NVivo (version 14; Lumivero). The codes were revised by the first authors and the second author (LK) to ensure reliability and to provide the option of adding newly identified codes when appropriate. The codebook consisted of 25 unique codes with a matching description (Multimedia Appendix 2). First authors JK, EM, and MSR have BSc degrees in health informatics and have experience in using qualitative and quantitative methods during their studies. JK, EM, and MSR were supervised by LK and RT, who have experience in using qualitative methods. ChatGPT (GPT-3.5; OpenAI) and Grammarly (Grammarly Inc), which offers artificial intelligence–powered writing assistance, were used to support the translation of selected anonymous quotes, the interview guide, and the codebook to improve readability and language.

### Ethical Considerations

Verbal and written information about the study was provided to all informants, and written consent was obtained from all of them. The study was conducted in accordance with the Helsinki Declaration. According to Danish regulations, health science questionnaire surveys and interview studies that do not involve human biological material (section 14(2) of the Danish Act on Committees) do not require reporting to, or approval from, the Danish National Centre for Ethics [30]. All data are stored in accordance with Danish legislation (General Data Protection Regulation). The informants were not reimbursed for their participation.

## Results

### Informant Characteristics

A total of 15 informants (n=9, 60% women; n=6, 40% men) were included. Their mean age was 80 (range 71-87, SD 5.3) years. Of the 15 informants, 12 (80%) had experienced falls within the last year, and 3 (20%) had experienced balance issues and dizziness. Other characteristics of the informants are summarized in Table 1, including sex, age, cohabitating status, and highest educational level attained.

**Table 1:** Informants’ characteristics.

ID	Sex	Age (y)	Living alone or cohabiting	Highest level of education attained^a^
#1	Female	81	Cohabiting	Medium
#2	Male	72	Cohabiting	Short
#3	Female	85	Living alone	Medium
#4	Female	80	Living alone	Medium
#5	Female	86	Living alone	Short
#6	Female	80	Cohabiting	Short
#7	Male	83	Cohabiting	Medium
#8	Female	87	Cohabiting	Comprehensive school
#9	Female	79	Living alone	Medium
#10	Female	71	Living alone	Medium
#11	Female	87	Living alone	Short
#12	Male	84	Living alone	Medium
#13	Male	74	Cohabiting	Long
#14	Male	75	Cohabiting	Short
#15	Male	80	Cohabiting	Short

^a^The education variable is aggregated from the 8 levels of the International Standard Classification of Education 2011 [31] and classified into 4 categories as follows: comprehensive school (typically up to lower secondary education), short education (including upper secondary and some postsecondary programs), medium education (encompassing bachelor’s and master’s degrees), and long education (referring to doctoral studies).

The findings are presented in four categories: (1) the user’s knowledge, skills, and experience with eHealth; (2) the user’s self-management; (3) the user’s social context; and (4) the user’s perception and mindset.

### The User’s Knowledge, Skills, and Experience With eHealth

There was a large diversity in how experienced the informants were with technology and how often they used it in their everyday lives. The informants were divided into three groups based on their knowledge, skills, and experience with technology: (1) those who used technology daily (experienced informants); (2) those who used technology to some extent (partially experienced); and (3) those who had limited use of, and skills with, technology (inexperienced informants). More than half of the informants (8/15, 53%) were experienced users of technology, and 5 (83%) of the 6 male informants were experienced users of technology. The experienced informants were frequent internet searchers and daily social media users. The partially experienced informants only used technology for necessary purposes, such as checking email. Finally, the inexperienced informants had, to some extent, given up on technology either because they had no interest in using it or because they found it too difficult to use; these were also the oldest of the informants.

The experienced informants were aware of the fluctuating credibility of web pages on the internet:

It must be kind of random what you can do, what you find, and how you’re loaded with the information you’re seeking. So, you can risk finding something, I wouldn’t say a lie, but something that might not be what you were searching for.

Informant #2, male, aged 72 years

All informants had mobile phones, and most of them (14/15, 93%) also had computers. The informants, usually the experienced ones, had smartphones (11/15, 73%), while some had nonsmartphones (4/15, 27%). The evolution of technology was unwelcome to some of the informants, and they were more comfortable using familiar technologies:

I don’t have a modern cell phone, nor do I want one. I have this old Nokia, and it can send a text [message] and tell me what time it is; I can forward my landline calls to it whenever I’m out for a walk, so I just have it with me in my pocket.

Informant #3, female, aged 85 years

It’s [the cell phone] just this one you can call from. It [the cell phone] can only do what I need it to do.

Informant #11, female, aged 87 years

Several informants used health technologies, such as pedometers, click-ons for hearing aids, and health information web pages. Experienced users were the ones who used technology to search for health information, but only a few used the Danish national health portal:

It was not long ago that I was on it [the Danish National health portal] to see all my diseases and look at my test results.

Informant #7, male, aged 83 years

### The User’s Self-Management

The data indicate that the informants in general were interested in taking care of their health, and several were making efforts to do so, including being physically active, but seemingly they paid limited attention to their nutritional intake. One informant expressed how important it was for her to be physically active for fear of losing the ability to move around freely one day. The same informant mentioned that she does not pay much attention to what she eats, arguing that she feels good now, so what she is eating must be fine:

Can you tell me about how you try to take care of your health?

Interviewer

Well, first and foremost, I do that by being physically active. And I’m so scared actually, it’s something I’m afraid of, that one day I won’t be able to move anymore.

Informant #11, female, aged 87 years

Do you ever think about what you should eat in relation to your health?

Interviewer

Not so much. I must admit.... I tell myself that I feel so good and I can still do so much, so what I eat cannot be completely wrong.

Informant #11, female, aged 87 years

Many of the informants understood and adhered to the health information they received from the internet, their relatives, health care professionals, and other sources of information, whereas only a few informants searched for health-related information themselves. Those who searched for health information typically used the internet or consulted their friends and relatives. Seemingly, the informants living with chronic conditions were more aware of seeking and appraising health information to minimize the impact of their conditions:

And we [friend] talked a lot about what she did...Well, but she recommended the anti-inflammatory diet, and so I did that for a longer period.

Informant #4, female, aged 80 years

And specifically with Parkinson’s, you stiffen up, so it’s especially good to stay active.

Informant #9, female, aged 79 years

Regarding nutrition, it also seemed that primarily those informants with diet-related conditions either sought or received information from health care professionals. Some mentioned receiving and adhering to nutritional advice from their general practitioners to better manage their conditions, such as high cholesterol, imbalanced salt concentration, and celiac disease.

Several informants set health-related goals for themselves to stay physically active in their everyday lives, such as taking daily walks, achieving a certain number of steps, and losing weight. In addition, they focused on maintaining mental well-being. Two of the informants, whose goals were taking daily walks and achieving 10,000 steps a day, expressed how they used pedometers to track their progress:

We walked, well, over 10,000 steps per day.

Informant #1, female, aged 81 years

Some of the female informants had goals related to losing weight or avoiding weight gain. Several had tried various weight reduction diets that they had heard about from relatives or the media, including commercial television programs. Diets included the ketogenic diet and intermittent fasting. In general, all informants who had health-related goals focused on achieving these goals. Some also expressed how their goal of having positive attitudes toward physical activity helped them become more physically active in their everyday lives:

Then I have a good [motivational] phrase: I always tell myself that what I could do yesterday, I can also do today.

Informant #11, female, aged 87 years

### The User’s Social Context

In general, the informants’ relatives, particularly their children and grandchildren, were involved in, and supported them in, managing their health in terms of their nutritional intake and physical activity. Relatives inquired about the informants’ health and took the initiative to help them improve it. These initiatives were often in the form of encouragement and incentives to be physically active, such as walking or using workout equipment or exercise bikes:

[T]hen my daughter and my son-in-law came by last Sunday and asked whether I wanted to go for a walk, and so, of course, I went with them. That happens sometimes.

Informant #3, female, aged 85 years

ago that I can use at home.

Informant #12, male, aged 84 years

The informants expressed that their relatives also played a role in shaping their eating habits, such as by introducing them to new diets or suggesting nutritional changes. However, only a few informants expressed involvement from their relatives regarding nutritional intake (compared to involvement in their physical activities):

[My son and daughter-in-law] have switched to a vegetarian diet 3 times a week, and we are also on board with that.

Informant #2, male, aged 72 years

Friends were also important sources of support. For the informants, talking with friends about their disease was a way to feel supported by discussing and sharing their experiences with peers, whereas relatives were the most important sources of support when it came to encouragement and incentives to improve their health:

[W]e ache and wonder how much one can actually become afflicted, as it gradually hits you. So, naturally, we discuss it a bit [with friends].

Informant #9, female, aged 79 years

Relatives, particularly the informants’ children, were also perceived as important sources of support for technology use. They helped with the installation of new technologies and with other technical difficulties, such as pressing the wrong buttons or understanding how to use new apps:

[A]nd then maybe I’ve got a hold of something, and without knowing what it is, I fiddle and press various buttons, you know. And then it’s good that I have him [his son].

Informant #2, male, aged 72 years

Only one informant had neither a child nor a spouse, and she experienced less social support than the other informants. This informant had no interest in physical activity, did not receive incentives from others to improve her health, and did not talk to friends about health-related topics to avoid burdening them:

It’s not that I don’t have good friends. But now, for instance, I have a very good friend who has been sick, with ataxia, and I don’t want to burden her with that, you know. Because she’s very sick already, so I don’t want her to worry about me.

Informant #10, female, aged 71 years

If the informant had problems with technology, she sought help professionally, not from friends:

I have had some issues with my mail because YouSee and TDC [telecommunication providers] had some spam filters that hid all of my emails. I couldn’t enter my email then...then they wrote to me and told me how to fix it.

Informant #10, female, aged 71 years

Most of the informants had positive experiences with support from health care professionals from the geriatric outpatient clinic. They felt supported by the exercise programs provided by physiotherapists from the clinic. In many cases, the positive experiences of support were related to being provided with individualized physical exercises and advice relating to their health conditions:

And she [the physiotherapist] has shown me some exercises to help with my balance, among other things. And I’ve gotten one of those round cushions that are soft, you know, so I can stand and maintain my balance.

Informant #11, female, aged 87 years

However, a few informants felt less supported because they had to perform a large part of the physical exercises on their own at home, and they lacked detailed instructions for these exercises:

Sometimes, I feel like I need an instructor.

Informant #2, male, aged 72 years

It [exercise instructions on paper] doesn’t say; there are no measurements for the width of the board or how long you need to walk and how much you can deviate. I think it’s very unspecific.

Informant #7, male, aged 83 years

Most of the informants listened to, and followed, the advice provided by the health care professionals from the geriatric outpatient clinic regarding their health and how to minimize the risk of falling. The advice included drinking enough fluid during the day and using training equipment at home correctly. Despite the informants’ history of being at risk of falls, they generally did not report receiving information or advice from health care professionals regarding nutrition to maintain their physical function and thus minimize their risks of falling.

### The User’s Perception and Mindset

The informants’ physical activities varied and could be classified into three groups: (1) structured physical activities, (2) incidental physical activities, and (3) inactive. Approximately half (7/15, 47%) belonged to the group that engaged in structured physical activities, such as daily physical exercises or weekly planned exercises with peers [32,33]. One-third (5/15, 33%) belonged to the group that engaged in incidental physical activities, such as house cleaning, short walks, and gardening [33,34]. Only a few (3/15, 20%) belonged to the inactive group of informants, who only did what was necessary because they seemingly had little interest in physical activities.

The informants’ motivation for being physically active stemmed from the desire to remain physically mobile and avoid relying on others, as well as the opportunity for social interactions. Several informants found it enjoyable to participate in various structured physical activities together with their peers, such as going on excursions or joining training groups, rather than performing exercises alone:

And I’m so afraid; it’s actually one of the things I’m most afraid of, that one day, I can’t move, walk, or take care of myself anymore.

Informant #11, female, aged 87 years

And then I made a club down here, where we meet every Wednesday. And there [in the club], you’re motivated to engage in a lot of events through songs, or someone comes and gives lectures. We’d go for a walk in the forest, or take trips on a bus, where we have lunch, or take some trips in which we’d have lunch out. All such events.

Informant #8, female, aged 87 years

A few informants from the structured physical activities group described how they did not perceive themselves as physically active. Some even expressed that they were lazy:

I must say that I’m living with the effects of my broken shoulder. And I’m completely lazy.

Informant #4, female, aged 80 years

The informants from the incidental physical activities group were familiar with how performing daily physical activities improved their health, but despite this knowledge, their motivation to perform physical activities was limited. However, there was a tendency for social interactions to be motivating factors for the informants belonging to this group:

Yes, and I can say that I don’t think I’m fulfilling my responsibility [performing exercises] if I have to do it alone. I’m probably better at doing it with others.

Informant #8, female, aged 87 years

For the inactive informants, a primary reason for not exercising was the difficulty in finding personal meaning and purpose in physical activities. For some of these informants, the potential opportunity to engage in social interactions did not increase their motivation to be physically active:

I’ve always hated going on walks without a purpose. I’ve never played sports.

Informant #12, male, aged 84 years

Well, I don’t like to do anything when there are a lot of people around, and when I need to stand there and do one thing after another.

Informant #6, female, aged 80 years

Some informants who were physically impaired because of health-related conditions found it difficult to be physically active. These informants were also either in the inactive or incidental physical activity group:

Then, I’ll take a walk in the forest. I haven’t done that very much lately, perhaps because I’ve had an operation on my knee. I’m not particularly fond of it, but right now, I can’t go on my long walks.

Informant #3, female, aged 85 years

By contrast, the informants from the structured physical activities group had more knowledge about, and were more attentive to, staying active during and after an illness or injury.

Regarding the informants’ perception and mindset toward their food intake, many expressed uncertainty about whether there were particular foods that would benefit their health. However, the majority expressed that they should consume more vegetables, and some also expressed that they strived to do so. Vitamins, both as supplements and in food, were highlighted as important for staying healthy. One informant expressed that protein intake was beneficial for weight loss. No informant mentioned the importance of protein intake for maintaining muscle strength and thus preventing falls. Several informants expressed acceptance regarding not necessarily adhering to recommendations for healthy eating (generally referred to as the intake of vegetables and foods low in fat and sugar) because most of them were under the impression that they ate according to their nutritional needs:

I actually think I eat as I should.

Informant #3, female, aged 85 years

Despite the informants’ narratives revealing their perception of the concept of healthy food, it was evident that their food intake was largely guided by their preferences for the foods they liked. Some informants revealed positive attitudes toward allowing their preferences for tasty food to guide their intake:

I think about having some vegetables because it’s supposed to be good for the stomach. But otherwise, I don’t really think about whether it’s healthy or not. I think all food is healthy if you feel like it. Unless you overdo it, of course.

Informant #7, male, aged 83 years

Advanced age was also mentioned as a justification for not making any changes to their eating behavior just to adhere to the consumption of “healthy food”:

I suppose now that I’ve gotten so old, so...

Informant #6, female, aged 80 years

In general, the informants had a limited focus on their nutritional intake and on fulfilling their dietary needs. However, some female informants were more likely to report avoiding fatty food to avoid weight gain:

No, I don’t eat fatty food, I don’t eat butter on bread...It’s a waste of calories because you don’t need it; there’s liver pâté.

Informant #10, female, aged 71 years

Several informants perceived technology as a necessity in their daily lives; however, some expressed that they thought technology had too much of an impact on their lives:

And the worst part of it is this damn computer. It starts in the morning during breakfast when I open it up, and then I’ll check my email, then Ekstra Bladet [a Danish news magazine], and then Facebook, and only then am I ready for the day.

Informant #14, male, aged 75 years

Many of the informants had positive attitudes toward using a mobile phone because it made it easier for them to reach others and stay in contact with them. The experienced users preferred using computers or iPads because of their large screens, which made them easier to use:

Well, now if I should look at it [exercises on the computer] right, then I don’t have to deal with the small font. It’s nice with a big screen.

Informant #1, female, aged 81 years

Some informants expressed negative attitudes toward technology because they felt that it hindered personal communication and was challenging to manage. Several informants mentioned that negative experiences mainly occurred when the technology changed or did not work. These challenges were demotivating for the informants because it required time to resolve the problems and caused great frustration:

I think that, at least for us, there are too many times when it [the technology] doesn’t work...and it takes too long.

Informant #8, female, aged 87 years

## Discussion

### Principal Findings

We explored older adults’ use of everyday digital services and technology and how they acquire knowledge about, and manage, their nutritional intake and physical activity in relation to their health. A main finding in this study was the great diversity in the informants’ experiences, mindset, and use of technology: some perceived technology as a necessity in their daily lives, whereas others viewed it as a source of frustration. Feelings of frustration were more prevalent among the oldest informants. Another main finding was that, although the informants were at risk of falls and had been referred to a geriatric outpatient clinic for the assessment and management of their fall risk, they possessed limited focus on, and knowledge about, nutritional needs to promote or maintain good physical function and thereby decrease their risk of falling. An important finding was the positive impact of the social network of relatives and health care professionals on the informants’ use of technology and motivation for the self-management of nutritional intake and physical activity. There seemed to be a relationship between the informants’ levels of physical activity and having a positive mindset, whereby the most active informants seemed to be more aware of the benefits of staying active and thinking positive. By contrast, there was limited focus on the importance of their food intake, which seemed to be related to the informants’ limited knowledge about this topic and not to a lack of motivation to look after their health and well-being.

### Comparison With Other Work

The great diversity in the informants’ experiences and use of technology, with inexperienced informants belonging to the older age group, is also described in previous studies [6,35,36]. Rossen et al [35] examined readiness for technology and showed that the older age group had the lowest readiness for technology. However, the findings of Goyal et al [37] indicated that older adults may have greater adherence than younger adults once they adopt technology. This suggests that it is important to provide older adults with sufficient support to help them adopt technology. Although most of the informants (14/15, 93%) used technology, only a few (6/15, 40%) used it for health-related purposes. It was mainly the informants with chronic diseases (6/15, 40%) who searched for information on treatment and medication and generally focused on information that could minimize the impact of their chronic diseases. This is in alignment with 2 other studies and a recent review [6,23,38], which found that when older adults seek health-related information, it is often related to information about specific diseases, treatments, and medicines. Interestingly, those informants who used eHealth to find health-related information (6/15, 40%) mainly searched for information about their chronic diseases but did not search for information about how to prevent falls. This signifies that they do not consider information about preventive measures in relation to falls as being health related.

To understand individuals’ motivation and ability for self-management, it is important to gain insight into their perception and mindset because these affect their motivation to engage in a specific behavior [39,40]. Our finding that older adults generally have positive attitudes toward technology is supported by the results of other studies [6,41]. The main reason for not using technology was frustration with technological challenges and because it hindered personal communication. This aligns with the results in the review by Wilson et al [19], who found that barriers to using eHealth included dislike of the technology and problems with functionality. Perceiving technological challenges may affect individuals’ perception of ease of use, which is an important determinant for the intention to use technology [42]. Therefore, these potential barriers need to be addressed in future interventions. Furthermore, Wilson et al [19] found that a lack of knowledge and experience with using technology hindered use, whereas a belief in its benefits facilitated use. These findings are consistent with those of Terp et al [6], who found that the perception of technology as being useful facilitated its use among older hospitalized patients and that nonuse was mainly due to a lack of knowledge about the derived benefits from the technology. These findings suggest a need to provide older adults with knowledge about the advantages of using eHealth.

We found that the informants who were physically active (structured and incidental physical activities groups) were motivated to perform physical activities because they experienced these as being fun, they enjoyed the social element, they had been active throughout their lives, and they wished to remain physically mobile and avoid relying on others. These motivational factors for physical activity have also been described in other studies [43,44]. The reviews by Sandlund et al [43] and Bunn et al [44] found that important facilitators for commencing fall exercise programs were previous exercise habits, social support and interaction, the ability to remain independent, and the fun element. For the inactive informants, a primary reason for not exercising was the difficulty in finding personal meaning and purpose in physical activities. These findings also align with those of Sandlund et al [43] and Bunn et al [44], who found that the barriers to commencing fall exercise programs were lack of support and interest, concerns about the exercises, and unawareness of the benefits.

Our findings indicate that older adults, despite being at risk of falling, may have a limited focus on eating adequately to maintain or improve their physical health. Among several of the informants, the behavior and mindset toward food intake were focused on society’s notion of an ideal slim body, which corresponds to the findings of previous studies [26,45]. Our findings suggest that this may not be due to a lack of motivation but merely due to limited knowledge; the majority were motivated and focused on maintaining their health because of their fear of losing physical function. Despite the importance of adequate nutrition, previous studies have reported limited nutritional knowledge among older adults [45,46]. Our findings indicate that older adults are more likely to receive, rather than actively seek, health-related information, a result also supported by our previous study among Danish hospitalized patients [26]. Therefore, health care professionals play an important role in providing older adults with relevant information, including information on their nutritional needs and the risk of falling. Our data revealed that older adults, in general, trusted health care professionals and adhered to the advice they provided. Our findings indicate potential benefits in ensuring that older adults receive relevant information and advice in future fall prevention interventions.

Social support from family, especially from their adult children, had a positive impact on the informants’ technology use, self-management, and mindset concerning physical activity and nutritional intake. This positive influence on older adults’ technology use was reported in a review by Levin-Zamir and Bertschi [21], who found that social support is paramount for many older adults in executing tasks related to health information from media sources. This finding is also corroborated by Takemoto et al [41], who found that human support increases accountability and enhances the use of technological devices. The positive impact of support from the family on the informants’ mindset for nutritional intake and physical activity was also established in other studies [2,26,47]. Spiteri et al [47] reported that family support was one of the key motivators for physical activity among older adults, and Terp et al [26] found that relatives were an important resource for older adults’ food intake. The positive impact of social support on older adults’ self-management is also corroborated by Schnock et al [2], who found that being married or living with someone had a positive impact on engagement in fall prevention interventions and self-management among older adults. Although almost half of our informants (7/15, 47%) lived alone, our data showed that most of them (6/7, 86%) experienced social support. This is contradictory to the findings of another study [48] in which older adults who lived alone experienced less social support. Overall, in our study, relatives were seen to have a higher impact on the informants’ levels of physical activity than on their nutritional intake. This may be explained by a lack of knowledge among the relatives about the nutritional needs of older adults. In future fall prevention interventions, it is important that information about nutrition and its impact on physical function and the prevention of falls is also provided to relatives.

Several findings of this study correspond to those in our previous study, which we conducted among older hospitalized patients [6,26]. The population in the study reported in this paper differs because all informants were at risk of falling and underwent evaluation and treatment in a geriatric outpatient clinic. International guidelines [10] recommend fall prevention interventions that enhance health behavior to reduce the risk factors for falls, such as inadequate nutrition and a lack of physical activity. The informants were recruited from the outpatient clinic and had received information and training from the clinic before the interviews. We therefore expect that they were provided with information on risk factors and advice regarding optimal nutritional intake and physical activity. However, we cannot conclude from this study whether the informants had been offered such interventions. This study provides important knowledge from the perspective of older adults about their needs in terms of developing an eHealth-based intervention aimed at supporting and motivating better health behavior, thus preventing functional decline and its consequences, such as falls.

### Strengths and Limitations

A strength of this study was the use of READHY as a theoretical framework because it provided a conceptual understanding of relevant aspects of individuals’ readiness to engage with eHealth, such as digital health literacy, social support, and the capability to manage their own condition. The use of a modified version of the READHY framework with the addition of a fourth theme (perception and mindset) is better suited for a qualitative analysis process. Another strength is that the informants live in a country that is among the most digitalized in the world [49]. The use of digital services and technology is therefore common in the general population, and a lack of resources or difficulties in accessing the internet and eHealth are usually not barriers, as our data also indicate. Thus, as the lack of access to digital technology is not a barrier among this group of informants, it enabled us to explore how the informants acquire knowledge about their nutritional intake and physical activity in relation to their health. However, the transferability of the study is worth considering, given that the majority of the informants (11/15, 73%) lived in the same geographic area of Denmark (Nordsjælland). In future studies, the inclusion of informants from other geographic areas can help achieve greater heterogeneity.

### Conclusions

This study demonstrates the potential of eHealth to support self-management in older adults, as most of them already use digital technology in their everyday lives. Older adults’ age, social context, and mindset should be considered when implementing and supporting eHealth. They must be provided with knowledge about the benefits of using eHealth to improve their motivation to use it for the self-management of their nutritional intake and physical activity. Furthermore, health professionals must be aware of the need to educate older adults about the impact of nutritional intake and physical activity in fall prevention, particularly for those who lack social support.

## Appendix

Multimedia Appendix 1 Interview guide.

Multimedia Appendix 2 Codebook.

## Abbreviations

READHY: Readiness and Enablement Index for Health Technology
