# Supplementary material for: Knowledge, Skills, and Experience With Technology in Relation to Nutritional Intake and Physical Activity Among Older Adults at Risk of Falls: Semistructured Interview Study
Source: JMIR Hum Factors. 2024 May 8;11:e52575. doi: 10.2196/52575 (PMC11112469; doi:10.2196/52575)
Supplement: Multimedia Appendix 1 [file humanfactors_v11i1e52575_app1.docx]

| **Interview guide** | | |
| --- | --- | --- |
| **Introduction**  I would like to start by saying thank you for being a part of this conversation. My name is XXX, and I have XXX with me today, who is my fellow student, just listening and taking notes during our conversation. We are studying Health Informatics at the University of Copenhagen, and currently working on a major final project in collaboration with Gentofte Hospital. Our project focuses on how the elderly eat and move, exploring how technology can assist in these aspects. That's why we're eager to talk to you.  Whatever we hear and gather during this conversation will only be used in a way that ensures your anonymity. If there's anything you're unsure about or don't understand during the interview, feel free to ask. We expect the conversation to take about an hour. Is that okay with you? We'll be recording the conversation so that we can work with the information you provide. We'll delete the recording once the project is completed.  Enough about the practicalities; let's start the conversation. I'll go ahead and press record, and then we can get started. To begin, I can share a bit about myself.   *Provide information about yourself.* | | |
|  |  |  |
| **Semistructured interview** | | |
| **Main themes** | **Dynamic questions** | **Follow-up questions (check-up)** |
| Introductory questions | Can you tell me a bit about yourself?   - Do you suffer from any chronic illnesses? Are you currently taking any medication?   Have you experienced a fall in the past year that led to a hospital visit? | How old are you? Are you retired? What was your occupation?  Do you live alone? Are you married?   **If yes:** 1. Did you lose consciousness when you fell?  2. Do you have difficulties maintaining balance in your daily life?  3. Have you experienced multiple falls within the last year?  4. Do you experience dizziness in your everyday life?  **If no:** 1. Do you experience dizziness in your daily life?  2. Do you have difficulty walking and maintaining balance?  - Do you use any aids  when walking?  3. Have you come close to falling one or more times? What prevented you from falling?  4. Do you experience dizziness in your daily life? |
| Diet and exercise | Can you provide me with an example of what you typically eat during the week?     Can you give examples of any foods that are important for you to include in your diet for health reasons?  Can you tell me a bit about how it typically goes when it is time to cook dinner?    Can you try to describe to me what daily activities you engage in during a typical week?  What motivates you to move and be physically active? | Do you eat certain things frequently?   - What are your favorite foods? - Are there things you avoid?   Do you do anything specific to ensure you eat what you need?   - Do you have a meal plan? Who created it for you? - Do you keep track of how much you eat in a day? - Do you seek advice/help from others regarding what to eat? (family members/health professionals). Where?   Who decides what you will have for dinner?  Do you cook meals together?  Do you take walks? (possibly with someone)   - How often do you approximately go for a walk?   How do you get around to various activities throughout the day?  Do you enjoy being physically active?  **Check:**  Do you follow a specific workout/training plan? Or have you followed one before?   - Can you tell me a bit about that training plan? Do you exercise alone or with others? - How long have you been following the program? - What does/means the training to you in your daily life? |
| Self-management | What do you associate with the word 'health'?   - Can you give me a few examples of how you take care of your own health?   Where do you typically receive advice and information regarding health or general well-being? | Do you talk to others about your health?   - Do you receive assistance from family members, doctors, or others?   Have you ever used the internet to seek advice?   - Can you provide examples of places online that you have used? |
| Technology | Can you give me some examples of how and when you use technology in your daily life?                                   Do you use any technology related to your health? | **Use Technology:**  How often do you use technology?  What type of technology do you use? (tablet, mobile, or computer)  Do you own a computer/tablet/mobile phone?   - What have you used your computer/tablet/mobile phone for in the past week?   What do you think about using XX (phone/tablet/computer)?   - Can you provide an example of a situation where using XX (mobile/tablet/computer) helps you?   Have you faced any challenges in using technology? What are they?  **Use no Technology:** Is technology too difficult to use?  Have you ever used a computer or any form of technology?   - What were your experiences?   **If yes:**  What technology do you use?  In what situations do you use this technology?  Do you face any challenges with this technology? What are they?  **If no:**  Have you ever tried using technology to keep track of your health?  What is the reason for not using it? What challenges have you encountered before? |
| **Introduction to Helpii**  Have you heard about Helpii before? Helpii is a digital website designed to assist elderly individuals with health challenges in staying physically active. The website is structured with various courses tailored to specific health issues. These courses target older individuals with mild health concerns such as difficulty with walking. The courses consist of multiple short videos providing helpful advice and exercises to prevent falls. | | |
| Overview of Helpii  Chapter 2 - Understanding the Causes of Falls.  Chapter 13 - Nutrition: Insights into fall prevention. | What do you associate with the word 'skavank' (Eng: infirmity)?  Let me give you a quick tour of this website. What are your initial thoughts about the website?  (Show an overview of the topics on the site) | Are there any topics that you find particularly interesting? Which ones sound appealing to you?  Can you envision yourself using the site?  What would be important for you to have on the website if you were to use it? |
| Debriefing and conclusion | Thank you. Those were the questions I had for you. Is there anything you would like to add that you think is important to include?  Do you have any questions for us?  Thank you for the conversation and your time today. If any questions arise, feel free to reach out to us afterward. |  |
