# Supplementary material for: Knowledge, Skills, and Experience With Technology in Relation to Nutritional Intake and Physical Activity Among Older Adults at Risk of Falls: Semistructured Interview Study
Source: JMIR Hum Factors. 2024 May 8;11:e52575. doi: 10.2196/52575 (PMC11112469; doi:10.2196/52575)
Supplement: Multimedia Appendix 2 [file humanfactors_v11i1e52575_app2.docx]

| **Codebook** | | | |
| --- | --- | --- | --- |
| **Main categories** | **Sub-categories** | **Codes** | **Description** |
| The user’s knowledge, skills, and experience with eHealth | Experience with technology | Technology at home | What kinds of technology the informants has in their house |
|  | Knowledge, skills and experience with technology | Usage of technology | What does the user use the technology for |
|  |  | Can use technology | Can use technology without guidance |
|  |  | Can not use technology | Can not use technology without guidance |
| The user’s self-management | Health related goals | Health related goals and aspirations | Sets health related goals and aspirations |
|  |  | Focus on achieving goals | Working towards achieving the health related goals |
|  | Explore, understand and adapt to their own health situation | Seeks health questions | Seeks knowledge about health |
|  |  | Understand health information | Can understand the information they found and/or receive about their health |
|  |  | Adapts to health information | Use health informations to adapt to their own health situation |
| The user’s attitude and mindset | Attitude towards physical activity | Motivation to physical activities | Positive elements of physical activities |
|  |  | No motivation for physical activities | Negative elements of physical activities |
|  | Experience and attitude towards technology | Applicability identified | Identifies applicability of technology |
|  |  | Positive experiences | Feeling motivated to use technology and positive elements about technology |
|  |  | Negative experiences | Bad experiences with technology |
|  | Approach to nutrition | Nutritional inspiration | Seeks inspiration to nutrition |
|  |  | Attitudes towards nutrition | Attitude and thoughts of nutrition |
| The user’s social context | Involvement from relatives | Help with technology use | Relatives helps and guides in technology use |
|  |  |  |  |
|  |  | Health-related encouragement and help from relatives | Relatives listen and guides in relation to health |
|  | Support from health professionals | Physical activity and health incentives | Health professionals provides reason and motivation to perform health-related actions |
|  |  | Technology encouragement from health professionals | Healthcare professionals supports their instructions with technology |
|  |  | Lack of support | Lack of support from healthcare professionals |
|  |  |  |  |
|  | Follows instructions from healthcare professionals (health competence) | Training program | Have received a training program from healthcare professionals |
|  |  | Dietary guidelines | Have received dietary advice or a diet plans from healthcare professionals |
|  | Family and friends | Social interaction | How much social interaction means |
